# Fibroid vascularisation assessed with 3D Power Doppler as predictor for fibroid related symptoms and quality of life; a pilot study

**DOI:** 10.52054/FVVO.13.4.044

**Published:** 2021-12-30

**Authors:** A.L. Keizer, L.L. Niewenhuis, W.J.K. Hehenkamp, J.W.R. Twisk, H.A.M. Brölmann, J.A.F. Huirne

**Affiliations:** Department of Obstetrics and Gynaecology, Amsterdam UMC location VUmc, De Boelelaan 1117 1081 HV Amsterdam, the Netherlands; Department of Epidemiology and Biostatistics, Amsterdam UMC location VUmc, De Boelelaan 1117 1081 HV Amsterdam, the Netherlands.

**Keywords:** Uterine fibroid, 3D, power doppler, vascularity, heavy menstrual bleeding, health related quality of life

## Abstract

**Background:**

Uterine fibroids present differently, from well vascularised up to calcified, with some causing heavy menstrual bleeding (HMB).

**Objectives:**

To investigate the association between fibroid vascularisation and HMB, other fibroid related symptoms and quality of life (QOL).

**Materials and Methods:**

A single centre pilot study was carried out in the Netherlands. Women with a maximum of two fibroids who chose expectant management were included. 3D sonography including power doppler was performed at baseline and at 3, 6 and 12 months follow up. Women were asked to complete the Pictorial Blood Assessment Chart (PBAC) and Uterine Fibroid Symptom and Quality of Life (UFS-QOL) questionnaires at every visit.

**Main outcome measure:**

The association between fibroid vascularisation and HMB.

**Results:**

53 women were included in the study. Baseline fibroid vascularisation, measured as vascular index (VI) is associated with PBAC score; a 1% higher VI at baseline leads to an 11 point increase in PBAC score over time (RC 10.99, p=0.05, 95% CI -0.15 – 22.12). After correction for the baseline variables ethnicity and fibroid type the association becomes stronger (P<0.05). Fibroid volume at baseline and HMB are also associated: a 1 cm3 larger fibroid leads to 0.6 points increase in PBAC score over time (RC 0.56, p=0.03, 95% CI 0.05 – 1.07).

**Conclusions:**

This study highlights that both fibroid vascularisation and fibroid volume may be associated with an increase in menstrual blood loss, other fibroid related symptoms and QOL over time.

**What is new?:**

We used 3D power doppler to predict symptomatic fibroids.

## Introduction

Uterine fibroids can cause dysmenorrhea, (non- cyclic) pelvic pain, pressure symptoms, such as altered urinary frequency and defecation pattern, and problems with reproduction (Gupta et al., 2008; Stovall, 2001). Furthermore, fibroids have a negative impact on quality of life (Parker, 2007). Fibroids are also associated with heavy menstrual bleeding (HMB), although not all fibroids cause HMB (Wegienka et al., 2003). There is no consistent relationship between the size and location of fibroids and HMB (Parker, 2007). It has been reported that uterine fibroids are asymptomatic in at least 50% of cases. Contrarily HMB occurs in up to 30% of the symptomatic women (Gupta et al., 2008).

HMB and reproductive problems are related to the level of distortion of the uterine cavity, meaning these symptoms are more frequent in cases of submucous fibroids (Golan et al., 2001; Klatsky et al., 2008; Pritts et al., 2009). According to the FIGO PALM COEIN classification, submucous fibroids are types 0,1,2 and 3, intramural fibroids type 4 and subserosal fibroids types 5, 6 and 7. Fibroids that impact both endometrium and serosa are classified as type 2-5 (Munro et al., 2011). Fibroids present differently, from well vascularised up to degenerated or calcified non-vascularised. Vascularisation has been reported to be a predictor for fibroid growth (Nieuwenhuis et al., 2017). We hypothesise that besides the location of fibroids, size and vascularisation affect the degree of (heavy) menstrual bleeding. We expect that well vascularised fibroids will induce heavier bleeding than for example the same type of fibroids that are calcified or degenerated. We expect vascularity will not affect the bleeding pattern in case of subserosal fibroids (type 5, 6 or 7). In a previous publication we demonstrated that fibroid vascularisation is associated to fibroid growth over time (9). In this paper we used 3D power doppler (3D PD) for determining fibroid volume and vascularisation. 3D PD is reported to be a reproducible technique in the assessment of uterine fibroid volume and in the quantification of its vascularity (Minsart et al., 2012; Nieuwenhuis et al., 2015; Nieuwenhuis et al., 2017). Determining vascularisation and thereby predicting which fibroids will potentially give more complaints over time, could have implications for counselling patients on therapeutic options.

In this paper we investigate if fibroid volume and/ or the degree of vascularisation -quantified by 3D PD- is associated with HMB and other symptoms or quality of life. Secondly, we hypothesise that fibroid size at baseline is associated with symptom increase over time.

## Methods

### Study design

Data from a prospective cohort study were used. Data collection took place between March 2012 and March 2014 at our outpatient clinic, department of obstetrics and gynaecology, Amsterdam University Medical Centre (tertiary referral centre), Amsterdam, the Netherlands. All women diagnosed with a maximum of 2 fibroids without the use of hormonal drug therapy, who chose expectant management were consecutively asked to participate and included during the study period. Exclusion criteria were fibroids larger than visible with a vaginal probe (in general > 8 cm), more than 2 fibroids at baseline, presence of adenomyosis (diagnosed by ultrasound), current pregnancy and hormonal or surgical therapy planned / started within 12 months. A maximum of 2 fibroids at baseline was chosen to avoid any risk of mixing measurements of the fibroids in the same patient during follow up, women with additional fibroids discovered during follow-up were not excluded.

The study was listed in the Dutch Trial Register; number NTR3349 and approved by the ethical board of the Amsterdam UMC, location VUmc. All participants gave their written informed consent.

We performed ultrasonography and patients completed Pictorial Blood Assessment Chart (PBAC) (Hald and Lieng 2014; Higham et al., 1990; Janssen et al., 1995; Janssen, 1996; Reid et al., 2000; Zakherah et al., 2011) and Uterine Fibroid Symptom and Quality of Life (UFS-QOL) (Spies et al., 2002) questionnaires at baseline and after 3, 6 and 12 months of follow up.

The primary outcome measure was the association between fibroid vascularisation (VI) at baseline and change of the amount of menstrual bleeding (PBAC score) over time. Secondary outcomes measures are the association between VI at baseline and other fibroid related symptoms and fibroid related quality of life (UFS-QOL scores) over time during one year follow-up, the association between VI at baseline and PBAC and UFS-QOL at baseline as well as PBAC and UFS-QOL at 12 months of follow-up. We also performed the same analyses for fibroid volume at baseline and change of symptoms (PBAC and UFS- QOL scores) over time and the association between volume at baseline and symptoms (PBAC and UFS- QOL scores) at single measurement points (baseline and 12 months).

### Ultrasound & machine settings

2D sonography (at baseline and after 3, 6 and 12 months of follow-up) and 3D sonography including power doppler (at baseline) were performed using the Accuvix V10 ultrasound machine (Samsung- Medison, Seoul, South Korea). Gel infusion sonography (GIS) was performed in cases of uncertainty regarding fibroid type. All volumes were acquired by an experienced examiner (LLN) in a standardised way using a 3D vaginal probe (5-8 MHz) as previously published to result in the best reproducibility (Collins et al., 2012; Minsart et al., 2012; Nieuwenhuis et al., 2015; Raine-Fenning et al., 2008). Power doppler settings were set to at a fixed Gain at 50dB, Frequency 5-8 MHz, pulse repetition frequency 0.60 kHz, and wall motion filter low. A maximum fibroid size of 8 cm was accepted, as the penetration depth of the vaginal probe is not adequate to reliably measure the fibroid nor its vascularity, in fibroids larger than 8 cm. Size, location, FIGO classification of the fibroid were noted. In case of two fibroids, the largest was measured to avoid any risk of confusing measurements of fibroids in the same patient during follow up. A 3D PD volume was taken to assess fibroid volume and vascular parameters. Fibroid location and size were also drawn schematically to ensure correct follow up of the same fibroids over time.

### Off-line evaluation of the 3D Volumes

**Figure 1:**
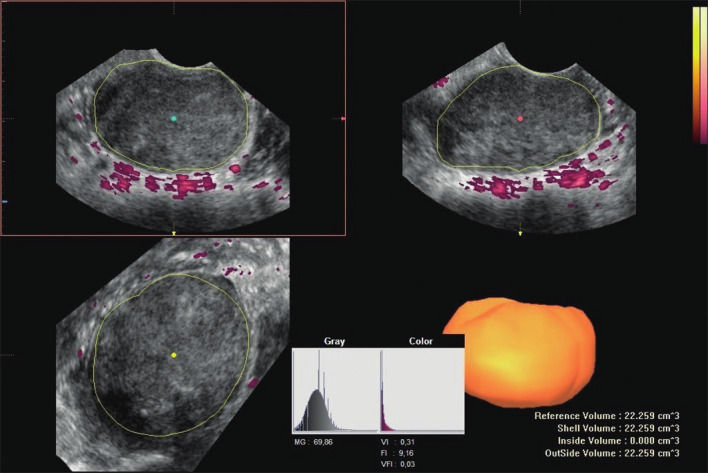
Offline analysis of power doppler indices automatically calculated using the histogram function.

All stored volumes were evaluated with VOCAL software, Sonoview Pro- 1.6.2. (Samsung-Medison, Seoul, South Korea). 3D sweep quality was scored 1 to 5 on a Likert scale for different US entities (1. contrast, sharpness, brightness, 2. visibility of fibroid (border), 3. penetration depth, 4. total fibroid visible in sweep, 5. movement artefacts). Volume and Vascular Index (VI) were calculated using the manual contour mode in VOCAL (Virtual Organ Computer-aided AnaLysis). Fibroid contours were drawn in six consecutive planes using a 30° rotation step. Power doppler indices were then automatically calculated using the histogram function. Fibroid contours measured did not contain its capsule. The fibroid capsule was measured separately, for this is seen as a separate entity (Nieuwenhuis et al. 2015). In short, this was either done in automatic mode or manually by visualising the often dense rim and using flow. The VI represents the proportion of blood vessels within the tissue (number of colour voxels divided by the total number of both colour and grey voxels) (Figure 1).

### Questionnaires

Patients were asked to fill out a Pictorial Blood Assessment Chart (PBAC) to quantify menstrual blood loss (Hald and Lieng, 2014; Higham et al., 1990; Janssen et al., 1995; Janssen 1996; Reid et al., 2000; Zakherah et al., 2011) and Uterine Fibroid Symptom and Quality of Life questionnaire (UFS- QOL) (Spies et al. 2002) at baseline and after 3, 6 and 12 months of follow up. A PBAC score of ≥150 was considered to represent heavy menstrual bleeding (Janssen et al., 1995; Janssen, 1996; NVOG, 2013; Zakherah et al., 2011). The UFS-QOL symptom score is based on complaints of abnormal and heavy menstrual bleeding as well as bulk symptoms. UFS- QOL symptom scores range from 0-100 (0 meaning no symptoms) and UFS-QOL quality of life scores range from 0-100 (100 meaning best possible quality of life).

### Statistical analysis

All analyses were performed using IBM SPSS Statistics 25.0 software package (IBM, New York, NY, USA). Linear mixed model analyses for repeated measurements were applied to analyse baseline fibroid vascularisation in relation to fibroid related symptoms (PBAC, symptoms and health related quality of life) over one year of follow up, which could also account for any missing questionnaires. Analyses were adjusted for fibroid volume at baseline and also for ethnicity, number of fibroids and fibroid type. Secondly the relationship between fibroid volume at baseline and the course of fibroid related symptoms during one year follow up was studied similarly. A p<0.05 was considered statistically significant. Linear regression was used for subgroup analyses, which were performed for type of fibroid (submucous, intramural and subserosal) and number of fibroids (1 vs >1). The subgroup of submucous fibroids was defined as all women having at least one submucous fibroid.

## Results

### Patient characteristics

Between March 2012 and March 2014 436 women with symptomatic fibroids visited our outpatient clinic. Unfortunately, the majority of women (370 of 436) had already used medical therapy, started medical therapy within 3 months or underwent surgery within 3 months, had a high number of fibroids, had fibroids only visible with abdominal probe, had no fibroids, were diagnosed with adenomyosis, were postmenopausal or were not willing to participate (<5). The possibility of starting medical or surgical treatment at follow up was discussed with all women. 78 of the 436 women were eligible and willing to participate. 66 of the 78 women were followed with 3D PD ultrasound for one year, 12 were lost to follow up. Of the 66 women, 53 women (80%) filled out one or more PBAC or UFS-QOL questionnaires and could be included in our analyses. In the course of follow-up 3 women were pregnant, 1 women had a miscarriage and 1 woman started ICSI. Women were included in the analysis when they had at least 2 measurements between which no hormonal medication was used or no pregnancy occurred. When hormones were started hereafter, or when someone fell pregnant, further measurements were excluded. During follow-up 7 women developed more than 2 fibroids, they were not excluded. Patient and fibroid characteristics are summarised in Table I.

**Table I:** Patient and fibroid characteristics.

Age (mean, SD)	43.4 (7.2)		
Ethnicity	European		28 (52.9%)
	African		8 (15.1%)
	South-American		8 (15.1%)
	Asian		7 (13.2%)
	Other		2 (3.8%)
Parity	Mean 1.1 (SD 1.0)		
Number of fibroids	1 fibroid		27 (50.9%)
	≥2 fibroids		26 (49.1%)
Presenting symptom	Heavy bleeding		17 (32.1%)
	Pain		10 (18.9%)
	Bulk symptoms		4 (7.5%)
	Fertility problems		2 (3.8%)
	Combination		10 (18.9%)
	No complaints		10 (18.9%)
Number of fibroids	1		27 (50.9%)
	(>)2		26 (49.1%)
Single fibroids	Largest diameter < 5cm		20 (74.1%)
	Largest diameter > 5cm		7 (25.9%)
	Type	Submucous	4 (14.8%)
		Intramural	9 (33.3%)
		Subserosal	14 (51.9%)
Two fibroids	Largest diameter < 5cm*		12 (46.2%)
	Largest diameter > 5cm		14 (53.8%)
	Type**	Submucous	1 (3.8%)
		Intramural	16 (61.5%)
		Subserosal	9 (34.6%)

*Largest diameter of the largest fibroid **Type of most protruding fibroid

### PBAC score

Of the 53 women who filled out a questionnaire, 47 (88.7%) filled out one or more PBAC’s. The majority of patients experienced HMB (i.e. PBAC score > 150) (Table II).

**Table II:** PBAC and UFS-QOL scores.

PBAC score >150* at least once	All women	34 (72.3%)
	1 fibroid present	17 (68.0%)
	multiple fibroids present	17 (77.3%)
PBAC score consistently < 150		12 (25.5%)
Mean PBAC score at baseline	submucous fibroids	326 (SD 105.7)
	intramural fibroids	274.3 (SD 262.5)
	subserosal fibroids	1.7 (SD 89.0)
Mean symptom score baseline (0-100)**	All women	41.9 (SD 19.8)
	1 fibroid present	45.1 (SD 16.2)
	multiple fibroids present	38.8 (SD 22.7)
	submucous fibroids	49.4 (SD 25.0)
	intramural fibroids	40.9 (SD 21.45)
	subserosal fibroids	40.9 (SD 15.3)
Mean QOL score baseline (0-100)***	All women	62.7 (SD 19.1)
	1 fibroid present	58.1 (SD 15.8)
	multiple fibroids present	67.6 (SD 21.3)
	submucous fibroids	53.9 (SD 20.1)
	intramural fibroids	64.8 (SD 18.5)
	subserosal fibroids	61.9 (SD 20.4)

*Largest diameter of the largest fibroid **Type of most protruding fibroid

### Questionnaires; fibroid related symptoms and quality of life

53 women (100%) filled out one or more UFS-QOL questionnaires. Women with submucous fibroids seem to have more symptoms and lower quality of life (Table II).

### Fibroid vascularisation and heavy menstrual bleeding

The association between baseline vascularisation and menstrual bleeding over time (repeated measurements analysis) showed that a 1% higher VI at baseline resulted in a 11 point higher PBAC score over time, this result is borderline statistically significant (p=0.05), but becomes significant when adjusted for ethnicity or fibroid type (Table III). VI at baseline and PBAC score at a single measurement points (both at baseline and at 12 months) and subgroup analyses for type of fibroid showed no statistically significant association (Table IV).

**Table III:** Repeated measurement analyses for fibroid related symptoms over time.

Fibroid vascularisation (VI) at baseline and symptoms					Fibroid volume at baseline and symptoms		
		R	P value	95% CI	R	P value	95% CI
PBAC score over time		10.99	0.05	-0.15 - 22.12	0.56	0.03	0.05 - 1.07
	corrected for ethnicity	11.35	0.05	0.09 - 22.60	0.65	0.02	0.12 - 1.19
	corrected for type of fibroid	11.45	0.04	0.25 - 22.65	0.57	0.03	0.06 - 1.08
	corrected for number of fibroids	11.04	0.05	-0.21 - 22.30	0.56	0.03	0.05 - 1.08
	corrected for fibroid volume at baseline	10.15	0.06	-0.82 - 21.12	0.53	0.04	0.02 - 1.03

**Table IV:** Vascularisation, volume and fibroid related symptoms at single measurement points.

Fibroid vascularisation (VI) at baseline				Fibroid volume at baseline		
	Regression coefficient	P value	95% CI	Regression coefficient	P value	95% CI
PBAC at baseline						
Submucous fibroids	-50.65	0.71	-1351.20 - 1249.90	-2.89	0.19	-13.97 - 8.19
Intramural fibroids	-2.79	0.84	-32.71 - 27.13	0.99	0.21	-0.64 - 2.63
Subserosal fibroids	-15.46	0.41	-55.87 - 24.96	-0.46	0.50	-1.89 - 0.98
PBAC after 12 months						
Submucous fibroids	-367.09	0.63	-7415.48 - 6681.31	-.077	0.96	-146.50 - 144.96
Intramural fibroids	11.12	0.30	-11.86 - 34.10	1.43	0.001	0.80 - 2.06
Subserosal fibroids	3.67	0.90	-70.98 - 78.32	-1.05	0.10	-2.40 - 0.29
UFS-QOL symptom score at baseline						
Submucous fibroids	-18.31	0.64	-163.80 - 127.17	-0.69	0.54	-4.70 - 3.32
Intramural fibroids	0.16	0.86	-1.61 - 1.92	0.027	0.65	-0.10 - 0.15
Subserosal fibroids	-0.11	0.92	-2.37 - 2.14	-0.018	0.82	-0.19 - 0.15
UFS-QOL symptom score after 12 months						
Submucous fibroids	-106.78	0.22	-602.14 - 388.58	-1.27	0.55	-20.31 - 17.77
Intramural fibroids	-0.03	0.98	-3.36 - 3.29	0.184	0.003	0.08 - 0.29
Subserosal fibroids	-9.57	0.26	-28.51 - 9.37	0.084	0.46	-0.18 - 0.34
UFS-QOL QOL score at baseline						
Submucous fibroids	4.51	0.90	-126.68 - 135.69	0.88	0.30	-1.87 - 3.62
Intramural fibroids	0.20	0.79	-1.34 - 1.74	-0.007	0.90	-0.11 - 0.10
Subserosal fibroids	-1.17	0.35	-3.79 - 1.46	0.04	0.68	-0.16 - 0.24
UFS-QOL QOL score after 12 months						
Submucous fibroids	100.11	0.44	-957.62 - 1157.84	0.79	0.77	-25.97 - 27.55
Intramural fibroids	-0.43	0.79	-3.89 - 3.03	-0.05	0.50	-0.22 - 0.12
Subserosal fibroids	0.07	1.00	-26.66 - 26.80	0.12	0.31	-0.16 - 0.40

### Fibroid vascularisation and fibroid related symptoms and health related quality of life

The association between VI at baseline and UFS- QOL scores during 12 months follow up (repeated measurements analysis) were not statistically significant, see table III for results. No association was found for VI at baseline and UFS-QOL scores at single measurement points (table IV). Subgroup analyses for type of fibroid also showed no association.

### Fibroid volume and heavy menstrual bleeding

Fibroid volume at baseline and HMB over time (repeated measurements analysis) are associated; a 1 cm3 larger fibroid volume at baseline is associated with a 0.6 point higher PBAC score over time (p=0.03) (Table III). No association was found for baseline volume and PBAC score at single measurement points, except for intramural fibroids (Table IV).

### Fibroid volume and fibroid related symptoms and health related quality of life

A 1 cm^3^ larger fibroid volume at baseline is associated with a 0.06 higher UFS-QOL symptom score (scale 0-100) on average over time (p=0.03), however no association was found for UFS-QOL quality of life (Table III). An association at single measurement points was only found for baseline volume and UFS-QOL symptom score after 12 months of follow-up (table IV).

## Discussion

### Main findings

The results of the present study generally support our hypothesis that the degree of fibroid vascularisation -measured by 3D PD- is related to deteriorating symptoms over time (during 12 months follow up). Both fibroid vascularisation and, to a lesser degree, fibroid volume are associated with an increase of the amount of menstrual blood loss and fibroid related symptoms over time. A 1% increase in fibroid vascularisation at baseline leads to an 11 point increase in PBAC score over time, which can quickly amount to heavy menstrual bleeding problems (PBAC score > 150).

### Strengths and limitations

This is the first study comparing vascularisation and fibroid related symptoms in a longitudinal setting with repeated measurements. We used validated tools to measure our outcomes. One experienced examiner performed all ultrasound and offline measurements, excluding the risk of inter-observer variation concerning the ultrasound findings. Applied techniques to measure 3D volume and vascularity were tested in a previous study that determined the optimal settings in fibroid measurement (Nieuwenhuis et al., 2017). A maximum fibroid size of 8 cm was accepted. In large fibroids colour signals tend to disappear once exceeding a certain focal depth. This causes an underestimation of the fibroid’s vascularity. To exclude possible external influences on fibroid vascularisation, only women without hormonal or surgical therapy were included. We adjusted for several possible confounders and performed subgroup analyses. However, this selection has some shortcomings. It resulted in a small sample size, hampering the power of our study. We also included women with more than one fibroid. This can make interpretation of data more hazardous. Ideally only women with one fibroid would have been included and stratified according to fibroid type. By excluding women that received surgical therapy, we selected a group of women with relatively mild symptoms. This group is of interest because one would want to be able to predict the occurrence of symptoms in order to start treatment early or refrain from treatment. Excluding patients who chose therapy (thus with more complaints) could explain that in our study we did find a borderline statistically significant association between vascularity at baseline and an increase in PBAC score over time. Women with more symptoms and submucous fibroids most probably chose some sort of treatment. Another explanation may be that submucous fibroids, irrespective of vascularisation, already cause more complaints (due to their location) and vascularisation is of less influence in this type of fibroid. The relatively limited number of women in this group could have played a role as well. Given our selection, the results cannot be extrapolated to women receiving therapy or women with an extensive number of fibroids.

### Interpretations

Many theories are reported as to why fibroids cause HMB. It is hypothesised that an enlarged endometrial surface area with fragile vessels causes bleeding problems (Patterson-Keels et al., 1994; Stewart and Nowak, 1996). Another theory suggests that a reduced myometrial contractility, due to the presence of fibroids, causes bleeding problems. Several observational studies demonstrate a causal relationship between fibroids and abnormal uterine bleeding. Women with abnormal bleeding were found to have a higher prevalence of fibroids than asymptomatic women and conversely women with fibroids were found to have an increased risk of HMB and other symptoms like dyspareunia and non-cyclic pelvic pain compared to women without fibroids (Clevenger-Hoeft et al., 1999; Lippman et al., 2003; Marino et al., 2004; Wegienka et al., 2003). The clinical consensus among gynaecologists is that submucous fibroids are associated with HMB. This was confirmed in our population: women with submucous fibroids had the highest PBAC scores at baseline, the highest symptom score and lowest QOL score, but we had no power to reach statistical significance. Besides this, we found that fibroid vascularisation and fibroid volume were associated with PBAC increment over time.

The relation between fibroid volume and symptoms is supported by a previous study that reported fibroid size was the main factor to be associated with bleeding symptoms (2), although others found no association (28-30). Risk of heavy menstrual bleeding, without other fibroid specific symptoms, increased with size of fibroids (but were not associated with type of fibroid) (Wegienka et al., 2003).

Previous studies that assessed fibroid vascularity and symptoms used different methods to assess vascularity, like flow velocity or the presence of a leiomyoma artery, sometimes taking leiomyoma volume or uterine weight into account (Farmakides et al., 1998; Tsiligiannis et al., 2013; Tsuda et al., 1998). These studies report that vascularity of leiomyoma can be useful as a predictor of leiomyoma growth. They report little about the relation of vascularity and symptoms. These studies also used different methods to assess symptoms, from non-validated questionnaires (Tsiligiannis et al., 2013; Tsuda et al., 1998) to the alkaline hematin method to assess HMB (Farmakides et al., 1998). Only one study had follow-up moments every three months during a year (Tsuda et al., 1998).

In this study, health related quality of life scores were not significantly associated with vascularity nor volume at baseline. This might be explained by the responsiveness of QOL for fibroid symptoms: QOL is determined by symptoms but also other factors such as general quality of life or life-events.

## Conclusions

This pilot study shows that both fibroid vascularisation and fibroid volume at baseline are associated with an increase of the amount of menstrual blood loss and other fibroid related symptoms over time. These results are promising and encourage future studies to confirm that assessing fibroid vascularisation and volume using 3D PD ultrasound could potentially be used in the future for predicting symptoms, opting for treatment or expectative management and determine a follow-up interval. 3D PD can easily be performed in clinical practice but should not be implemented before future studies (in a more heterogeneous group of patients with fibroids) confirm its validity. More research in larger studies is needed to help understand and predict fibroid growth and fibroid related symptoms, enabling correction for all possible relevant predictive factors including fibroid type, ethnicity, age and parity.
